# The Disrupted Bidirectional Regulation and Coupling of Resting‐State Blood Pressure and Heartbeat in Hypertension

**DOI:** 10.1111/acel.70338

**Published:** 2025-12-26

**Authors:** Xin Jiang, Haoru Li, Huixia Ren, Meige Liu, Dan Zhou, Shuisheng Lin, Zongchen Liu, Jiawen Yuan, Nana Xie, Alan A. Cohen, Sen Pei, Junhong Zhou

**Affiliations:** ^1^ Department of Geriatrics Shenzhen People’s Hospital (The First Affiliated Hospital, Southern University of Science and Technology, The Second Clinical Medical College, Jinan University) Shenzhen China; ^2^ Guangdong Provincial Clinical Research Center for Geriatrics, Shenzhen Clinical Research Center for Geriatrics, Department of Geriatrics Shenzhen People’s Hospital Shenzhen China; ^3^ Department of Pharmacology Vanderbilt University Nashville Tennessee USA; ^4^ Hebrew SeniorLife Hinda and Arthur Marcus Institute for Aging Research Roslindale Massachusetts USA; ^5^ Jinan University Guangzhou China; ^6^ Robert N. Butler Columbia Aging Center, Mailman School of Public Health Columbia University New York New York USA; ^7^ Department of Environmental Health Sciences, Mailman School of Public Health Columbia University New York New York USA; ^8^ Division of Gerontology Beth Israel Deaconess Medical Center Boston Massachusetts USA; ^9^ Harvard Medical School Boston Massachusetts USA

**Keywords:** arterial stiffness, baroreflex feedback, blood pressure, heartbeat, hypertension, transfer entropy, walking performance

## Abstract

The bidirectional non‐linear communication between blood pressure (BP) and heartbeat is critical to cardiovascular homeostasis, which remains poorly understood, especially under hypertensive conditions. We implemented transfer entropy (TE), an information‐theoretic measure of directional coupling, to characterize such bidirectional coupling between BP and heartbeat and its relationships to arterial stiffness and walking performance in older adults. A total of 493 older adults (201 normotensive (NTN), 168 controlled‐hypertensive (controlled‐HTN), and 124 uncontrolled‐HTN) completed simultaneous recordings of resting‐state beat‐to‐beat BP and R‐R interval for ≥ 10 min. The TE from BP to RR (i.e., BP‐RR) and from RR to BP (RR‐BP) was quantified. Participants then completed the assessments of arterial stiffness (i.e., brachial–ankle pulse wave velocity, baPWV) and walking speed in single‐ and dual‐task conditions. The validation using surrogate data confirmed the physiological significance of TE (*p* < 0.0001). Both BP‐RR and RR‐BP TE were significantly lower in controlled‐ and uncontrolled‐HTN compared to NTN (*p* < 0.03). In NTN and control‐HTN, higher BP‐RR and/or RR‐BP TEs were associated with slower walking speed (*β* = −0.25 to −0.16, *p* < 0.04). Higher BP‐RR TE was associated with lower baPWV (*β* = −0.17 to −0.16, *p* < 0.04), while higher RR‐BP TE was associated with greater baPWV (*β* = 0.17–0.21, *p* < 0.03). No such significant associations were observed within uncontrolled‐HTN. The observations suggested that TE captures hypertension‐related disruption of bidirectional BP‐heartbeat information flow, reflecting impaired baroreflex feedback, exaggerated feedforward cardiac influence, and dampening with anti‐hypertensive therapy. The distinct associations with vascular stiffness and walking performance suggest TE as a promising marker of cardiovascular integrity and functional reserve in aging.

## Graphical abstract

The bidirectional information flow between blood pressure (BP) and heartbeat, as captured by transfer entropy (TE), is disrupted in hypertensive older adults, reflecting altered feedback and feedforward coupling. This BP–heartbeat TE is associated with distinct relationships to arterial stiffness and mobility, highlighting its potential to capture cardiovascular regulation and functional reserve.

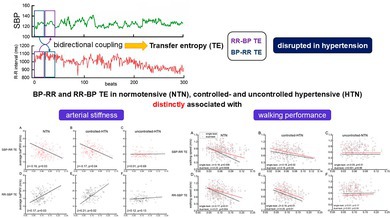

## 1 Introduction

The regulation of cardiovascular function is governed by dynamic interactions between blood pressure (BP) and heartbeat, mediated through both autonomic reflexes and vascular mechanics (Lanfranchi and Somers 2002). A well‐functioning cardiovascular system relies on the bidirectional feedback and feedforward communication between these elements (Javorka et al. 2017; Legramante et al. 1999). For example, the fluctuations in BP trigger compensatory adjustments in heartbeat via the baroreflex, while cardiac output influences vascular tone and pressure regulation (Lanfranchi and Somers 2002; Wehrwein and Joyner 2013). In the aging process, especially under the influences of cardiovascular conditions (e.g., hypertension), this delicate interplay is often disrupted, contributing to impaired autonomic control, arterial stiffening, and functional decline in hypertensive (HTN) older adults.

Traditional approaches to assessing cardiovascular regulation, such as baroreflex sensitivity or cross‐correlation between blood pressure and heart rate variability, have provided insights into autonomic control and vascular function (Monahan 2007; Rowley et al. 2006). However, these measures oftentimes simplify the complex, nonlinear, and directional interactions underlying the physiological coupling between blood pressure and heart rate, thereby overlooking subtle impairments in cardiovascular dynamics that may precede overt conditions (e.g., hypertension) or functional decline (Voss et al. 2009). This limitation is particularly consequential in the older adult population, in which cumulative vascular aging, stiffening of the arteries, and the high prevalence of hypertension disrupt the fine‐tuned interplay between autonomic and vascular systems (Ziegler et al. 2025). These changes not only increase vulnerability to cardiovascular events but also contribute to downstream consequences such as diminished mobility and increased fall risk. Therefore, novel non‐linear dynamic techniques are essential to more appropriately capture those early alterations in cardiovascular regulation.

Transfer entropy (TE) is an information‐theoretical technique that can quantify the direction and magnitude of information flow between two physiological procedures (Vicente et al. 2011). Unlike traditional measures, TE does not assume linear correlation between two signals. Several studies have shown preliminary evidence that altered TE between BP and heartbeat may indicate changes in cardiovascular regulation (Faes et al. 2011; Porta et al. 2000, 2017). For example, Faes and colleagues showed that in 12 healthy younger adults, the TE from BP to heartbeat was sensitive to a postural tilt stimulus (Porta et al. 2017). Still, the application of TE to large real clinical data of older adults remains limited; and more importantly, it is unclear how cardiovascular conditions (e.g., hypertension—whether controlled or uncontrolled) influence the bidirectional exchange of information between heartbeat and BP regulation, and how these alterations are related to the characteristics of cardiovascular system itself (e.g., arterial stiffness) and important functions (e.g., walking performance) in older adults.

Therefore, in this study, we used TE to characterize the bidirectional information exchanges between beat‐to‐beat BP and R‐R (RR) interval fluctuations of heartbeat based upon simultaneously recorded BP and RR data in normotensive (NTN), controlled‐HTN, and uncontrolled‐HTN older adults. We hypothesized that: (1) compared to NTN, both TEs from BP to RR (i.e., in the feedback direction) and from RR to BP (i.e., in feedforward direction) would be altered in both controlled‐HTN and uncontrolled‐HTN; and (2) the TEs would be associated with the degree of arterial stiffness and walking performance, and such associations would differ between cohorts.

## 2 Methods

### 2.1 Participants

This work was completed using the data from an electronic clinical repository/dataset maintained by the Department of Geriatrics, Shenzhen People's Hospital, Shenzhen, China. This repository was created with the goal to explore factors that pertain to cerebral‐ and cardio‐vascular function in older adults. All older adults who completed their general geriatric outpatient clinical visit since January 1st, 2022, were reached out to for potential recruitment to this ongoing repository; participants included in this study were those who had this visit before December 31st, 2024. The inclusion criteria of eligible participants were: (1) age ≥ 60 years at the first study visit, and (2) the ability to walk for at least 30 s without personal/physical assistance. The exclusion criteria were: (1) diagnosis of terminal disease (e.g., cancer); (2) hospitalization within the past 6 months; (3) diagnosis of overt neurological conditions (e.g., Parkinson's disease, stroke, or dementia); (4) chronic kidney disease and dyslipidemia; (5) other cardiovascular diseases (e.g., heart failure, coronary artery disease); (6) an inability to understand the study protocol; or (7) unwillingness to complete the study protocol. Older adults who were eligible for this repository then completed thorough assessments of clinical and functional characteristics.

The protocols of this repository were approved by the Institutional Review Board of Shenzhen People's Hospital and carried out in accordance with the guidelines of the Declaration of Helsinki. All participants provided written consent prior to participation in this study.

### 2.2 Study Protocol

In this dataset, participants completed three visits, that is, one in‐person screening visit, and if eligible, two study visits at the Department of Gerontology within the hospital. These visits were each separated by at least 24 h. On the first visit, eligible participants completed a series of questionnaires to assess demographics (e.g., age, sex, body mass index (BMI)), and duration of hypertension. One research staff administered these procedures. On the second study visit, participants completed a beat‐to‐beat finger BP assessment and measurements of arterial stiffness and walking performance. Two research staff administered this visit to ensure the quality of the assessment and the participant safety. Meanwhile, the study visits were completed before participants took any anti‐hypertensive medications. Participants were asked to refrain from eating or drinking caffeinated beverages 24 h before and throughout this study.

### 2.3 Hypertensive Characteristics

The hypertensive status of each participant was assessed according to their clinical record and examined on the screening visit. Specifically, hypertension was characterized as systolic BP (SBP) ≥ 140 mmHg and/or diastolic BP (DBP) ≥ 90 mmHg at the brachial artery of the left arm using an automated sphygmomanometer. We then categorized participants into normotensive (NTN), controlled‐hypertensive (controlled‐HTN) (i.e., those who were diagnosed with hypertension and actively taking related medication, yet whose SBP and DBP values were below the thresholds for hypertension) and uncontrolled‐hypertensive (uncontrolled‐HTN) (i.e., those who met the BP thresholds for hypertensive whether or not they were using anti‐hypertensive medication). We also recorded information on anti‐hypertensive medication use, including the medication type (i.e., calcium channel blockers (CCB), angiotensin‐converting enzyme inhibitors (ACEI), angiotensin‐II receptor blockers (ARB), beta‐blockers (BB), and diuretics) and the number of anti‐hypertensive medications participants used simultaneously. The number of anti‐hypertensive medications used and the duration of HTN were included as covariates in the following analyses.

### 2.4 BP and Heartbeat Recordings

On the second visit, each participant first completed the assessment of beat‐to‐beat BP series and simultaneously heartbeat series at resting‐state in a quiet assessment room. During the recording, participants were asked to avoid talking and refrain from moving as much as possible. Items that may interfere with the recording, such as mobile phones, were stored securely outside the room. Continuous beat‐to‐beat SBP and DBP series, and simultaneous heartbeat series were recorded using the Finometer PRO system (Finapres Medical Systems B.V., Netherlands) secured to the middle finger of the left hand with the participant lying supine (Guelen et al. 2008; Hodgson and Choate 2012; Park et al. 2025). The series was recorded for 10 to 15 min to ensure enough data points (as the number of beats per minute may vary across individuals). The sampling frequency was 100 Hz. The BeatScope software package (Finapres Medical Systems B.V., Netherlands) was then used to obtain the SBP and DBP series, and R‐R interval series. All these recordings consisted of at least 700 continuous sampled data.

### 2.5 Transfer Entropy

We followed the well‐established procedure of data preprocessing that has been used in previous studies (Park et al. 2025; Jiang et al. 2024). Then the pre‐processed series of 700 sampling points with the mean of zero were used for TE calculation to quantify the directed exchange of information between R‐R interval (RR) and BP regulation.

Theoretically, TE is an information‐theoretic measure that captures the degree to which the future state of one given process can be predicted by the past states of another process (the source), beyond what is already explained by its own past information. Uniquely, TE is without the assumption of linearity that is used in traditional linear correlation or Granger causality, and is sensitive to both linear and nonlinear dependencies (Vicente et al. 2011; Schreiber 2000).

Directional TE was calculated from time series X to time series Y using the following formula:

TEX→Y=∑py_t+1y_t^kx_t^llogpy_t+1y_t^kx_t^lpy_t+1y_t^k

where *y*_{*t* + 1} denotes the future state of *Y*, *y*_*t*^{(*k*)} the past *k* states of *Y*, and *x*_*t*^{(l)} the past *l* states of *X*. This formulation quantifies the reduction in uncertainty about *Y*'s future gained by incorporating past information from *X*.

The probability distributions were approximated using discretization‐based estimation of quantiles, and 33rd and 67th percentiles were used (Vicente et al. 2011; Kraskov et al. 2004). To determine the appropriate embedding dimension (i.e., the value of k and l) for the calculation of bidirectional TE between RR and BP (i.e., both SBP and DBP), we performed the computation following the recommendation as described in previous work (Porta et al. 2011). The specific procedure and results are presented in Supporting Information and Table S1. Based upon the results, we chose *k* = 3 and *l* = 3 for the calculation of RR‐SBP and ‐DBP TE, and SBP‐ and DBP‐RR TE (Porta et al. 2017, 2011; Lungarella et al. 2007). All these four TE outcomes were used in the following analysis.

To check if the obtained TE was of physiological significance, not due to random noise, we performed surrogate data testing. To test the null hypothesis of uncoupling while preserving the univariate dynamics of each signal, we constructed time‐shift surrogate data by circularly shifting the RR series by a random number from 50 to n‐50 (where n was the total length of the series) points, while keeping the SBP or DBP series unchanged (Faes and Porta 2015; Faes et al. 2008; Barrett et al. 2012; Quiroga et al. 2002). The lower bound of 50 beats was chosen to be much larger than any potential physiological interaction delay between RR and BP, which was typically within a few beats, thereby destroying genuine coupling while preserving the autocorrelation and amplitude distribution of each series. We computed TE on 5000 of this time‐shifted surrogate data for each relationship in each participant. When a significant difference between the original TE and surrogate TE was observed, it suggested that the observed directional dependencies between RR and BP exceeded those expected by chance.

### 2.6 Assessment of Arterial Stiffness

Arterial stiffness was assessed by measuring the left‐ and right‐side brachial‐ankle pulse wave velocity (baPWV) (Omron, Kyoto, Japan) (Turin et al. 2010; Yamashina et al. 2002). The average baPWV was then obtained by averaging left and right baPWV and used in the following analyses.

### 2.7 Walking Assessment

Following the arterial stiffness assessment, each participant completed the two trials of 10‐m walking (Peters et al. 2013) under each of the following conditions: walking quietly (i.e., single‐task condition) and walking while performing a verbalized serial‐subtraction‐of‐three task (i.e., dual‐task condition). They were asked to walk at their preferred speed along a 10‐m straight hallway, and a 20‐s resting period was provided between trials.

The walking speed was measured using the Mobility Lab system (Clario, Philadelphia, PA), consisting of six motion sensors to capture the kinematic data related to walking (Mancini et al. 2011). In dual‐task condition, a three‐digit random number was given by the study staff at the beginning of each trial, and the participant was asked to perform the serial subtraction throughout the trial. The walking speed averaged across two trials within each condition was used in the following analyses.

### 2.8 Statistical Analysis

Statistical analyses were performed with JMP 18 software (SAS Institute, Cary NC). The significance level of the analyses was set at *p* < 0.05. The normality of the data was examined using the Shapiro–Wilk test, and the homogeneity of variance was examined using the Levene's test. We first categorized participants into NTN and HTN, and then we further separated the HTN cohort into controlled‐HTN and uncontrolled‐HTN.

To compare the demographic and clinical characteristics between three groups, we used one‐way ANOVA models when data were normally distributed and the Kruskal‐Wallis test when the data were not normally distributed. Tukey's post hoc analysis was used to compare the factor means of significant models. For categorical variables (e.g., sex), we used a chi‐squared test. For the comparison between original TE and surrogate TE, we used non‐parametric Wilcoxon signed‐rank tests. We also assessed the associations between TEs and age, BMI, and sex by using linear regression models incorporating hypertensive status as a covariate (for age and BMI) and one‐way ANOVA models (for sex by comparing TE between women and men).

To test the hypothesis that the presence of hypertension would be associated with altered TE, we used one‐way ANOVA models when the data were normally distributed. The model factor was group (i.e., NTN, controlled‐HTN, and uncontrolled‐HTN), and the dependent variable was RR‐SBP and ‐DBP TE and SBP‐ and DBP‐RR TE in separate models. All models were adjusted for age, sex, BMI, duration of hypertension, and the number of anti‐hypertensive medication use, which were all believed to contribute to the cardiovascular regulation. Tukey's post hoc analysis was used to compare the factor means of significant models. When data were not normally distributed, we used the Kruskal‐Wallis test.

To test the hypothesis that TE would be associated with walking performance, we performed linear regression analyses for each group. Age, sex, BMI, duration of hypertension, and the number of anti‐hypertensive medications used were included as the covariates. Similarly, to examine the relationship between TEs and arterial stiffness (as assessed using average baPWV), we performed linear regression analyses as described above.

## 3 Results

After the search in the dataset, 201 NTN, 168 controlled‐HTN, and 124 uncontrolled‐HTN eligible older adults were enrolled and successfully completed all the assessments. All the outcomes were normally distributed with adequate homogeneity of variance. The demographic, clinical, and functional characteristics of each group are presented in Table 1. The one‐way ANOVA models showed significant differences in age, BMI, mean SBP and DBP levels, average baPWV, and walking performance (*p* < 0.006) between groups. Specifically, compared to the NTN group, controlled‐HTN and uncontrolled‐HTN groups had older age, greater BMI, higher average baPWV, and slower walking speed in single and dual‐task conditions. Mean SBP and DBP levels were greater in the uncontrolled‐HTN group compared to NTN and controlled‐HTN. No such significance was observed in the portion of women between groups (*p* = 0.97).

**TABLE 1:** The demographic, clinical and functional characteristics of participants.

		NTN (*n* = 201)	Controlled‐HTN (*n* = 168)	Uncontrolled‐HTN (*n* = 124)	*p* *
Age (years)		69.7 ± 7.4^A^	71.4 ± 7.6^B^	72.3 ± 7.9^B^	0.002
Sex					
Female (%)		102 (50.7%)	89 (52.9%)	67 (54.0%)	0.97
Male (%)		99 (49.3%)	79 (47.3%)	57 (46.0%)	
BMI		23.3 ± 3.4 ^A^	24.9 ± 3.6^B^	25.1 ± 3.8^B^	< 0.0001
Education (years)		10.2 ± 4.7	9.6 ± 4.9	9.4 ± 4.9	0.14
Average bapwv (m/s)		15.8 ± 2.8 ^A^	17.8 ± 3.5^B^	20.8 ± 4.6^C^	< 0.0001
Hypertension history (years)		N.A.	12.7 ± 8.9	11.1 ± 8.2	N.A.
Mean SBP (mmhg)		125.4 ± 9.3^A^	126.4 ± 9.1^A^	153.8 ± 9.6^B^	< 0.0001
Mean DBP (mmhg)		74.4 ± 6.4^A^	74.5 ± 7.2^A^	83.9 ± 9.8^B^	< 0.0001
Anti‐hypertensive medication (n)	CCB	n.a	108 (64.3%)	94 (75.8%)	n.a
	ACEI	n.a	9 (5.3%)	9 (7.3%)	n.a
	ARB	n.a	70 (41.6%)	48 (38.7%)	n.a
	BB	n.a	55 (32.7%)	41 (33.1%)	n.a
	Diuretics	n.a	13 (7.7%)	16 (12.9%)	n.a
Number of medications (n)	1	n.a	78 (46.4%)	66 (53.2%)	n.a
	2	n.a	63 (37.5%)	36 (29.1%)	n.a
	3	n.a	22 (13.1%)	18 (14.5%)	n.a
	4	n.a	5 (3.0%)	4 (3.2%)	n.a
	5	n.a	0 (0)	0 (0)	n.a
Walking speed (m/s)					
Single‐task		0.85 ± 0.21^a^	0.81 ± 0.21^b^	0.76 ± 0.22^c^	0.0002
Dual‐task		0.76 ± 0.23^a^	0.71 ± 0.22^b^	0.69 ± 0.24^b^	0.006
Transfer entropy					
SBP‐RR		0.068 ± 0.025^A^	0.062 ± 0.023^B^	0.059 ± 0.021^B^	0.002
DBP‐RR		0.072 ± 0.027^A^	0.065 ± 0.025^B^	0.066 ± 0.026^B^	0.02
RR‐SBP		0.094 ± 0.033^A^	0.085 ± 0.025^B^	0.087 ± 0.028^B^	0.03
RR‐DBP		0.116 ± 0.041^A^	0.103 ± 0.039^B^	0.104 ± 0.036^B^	0.01

*Note:* ^A,B,C^: different letters reflected where the significant difference was based upon the results of post hoc Tukey's test.

Abbreviations: baPWV, brachial‐ankle pulse wave velocity; BMI, body mass index; DBP, diastolic blood pressure; FMD, flow mediated dilation; HTN, hypertensive; NTN, normotensive; RR, R‐R interval; SBP, systolic blood pressure.

* *p* values were obtained by using one‐way ANOVA models when data were normally distributed and the Kruskal‐Wallis test when the data were not normally distributed. For categorical variables (e.g., sex), the chi‐squared test was used.

The surrogate data testing demonstrated that the TEs obtained from the original signal were significantly greater than those from the time‐shifted surrogate data (surrogate RR‐SBP TE = 0.034 ± 0.019; surrogate RR‐DBP TE = 0.032 ± 0.023; surrogate SBP‐RR TE = 0.021 ± 0.022; and surrogate DBP‐RR TE = 0.023 ± 0.023; all *p* < 0.0001). This suggests that the original TEs captured physiologically meaningful information exchange between the heartbeat and blood pressure regulation.

The exploratory linear regression models for the association between age and TEs showed that older age was significantly associated with lower BP‐RR TEs (*β* = −0.09 to −0.08, *p* = 0.03–0.04), and/or greater RR‐BP TEs (*β* = 0.12–0.14, *p* = 0.01); and no such significant association was observed between BMI and TEs (*r* = 0.01–0.08, *p* = 0.18–0.42). Additionally, no significant difference in TEs was observed between men and women (*p* = 0.16–0.66).

In the following sections, for ease of presentation, we only show TE between RR and SBP in the main text; the results of DBP were similar to those of SBP and presented in Supporting Information.

### 3.1 The Information Exchange Between SBP and Heartbeat Regulation

For the directional information exchange from SBP to heartbeat, the one‐way ANOVA models demonstrated significant effects of group on SBP‐RR TE (*p* = 0.002), and such effects were independent from age, sex, BMI, the duration of hypertension, and the number of anti‐hypertensive medications used. Specifically, the post hoc analysis revealed that compared to NTN group, the TEs in both controlled‐ (*p* = 0.0004) and uncontrolled‐HTN (*p* = 0.01) were significantly lower, and no significant differences between controlled‐ and uncontrolled‐HTN groups were observed (*p* = 0.37) (Table 1).

For the directional information exchange from heartbeat to SBP, the ANOVA models demonstrated significant effects of group on RR‐SBP TE (*p* = 0.01), and such effects were independent from age, sex, BMI, the duration of hypertension, and the number of anti‐hypertensive medications used. Specifically, the post hoc analysis revealed that compared to the NTN group, the TE in the controlled‐HTN group (*p* = 0.003), and in the uncontrolled‐HTN group (*p* = 0.03), was significantly lower; and no significant differences between controlled‐ and uncontrolled‐HTN groups were observed (*p* = 0.61) (Table 1).

### 3.2 The Association Between the TE to Walking Performance and Arterial Stiffness

Table 2 demonstrated the results of the linear regression analyses for the associations between SBP–RR TE and walking speed in single‐ and dual‐task conditions, and average baPWV.

**TABLE 2:** The association between transfer entropy between heartbeat and SBP and walking performance and arterial stiffness*.

	Walking speed		Average baPWV
	Single‐task	Dual‐task	
NTN			
SBP‐RR TE	*β* = −0.18, *p* = 0.009	*β* = −0.25, *p* = 0.0006	*β* = −0.16, *p* = 0.03
RR‐SBP TE	*β* = −0.17, *p* = 0.01	*β* = −0.21, *p* = 0.004	*β* = 0.17, *p* = 0.03
Controlled‐HTN			
SBP‐RR TE	*β* = −0.18, *p* = 0.02	*β* = −0.16, *p* = 0.04	*β* = −0.17, *p* = 0.04
RR‐SBP TE	*β* = −0.19, *p* = 0.01	*β* = −0.20, *p* = 0.01	*β* = 0.21, *p* = 0.02
Uncontrolled‐HTN			
SBP‐RR TE	*β* = −0.05, *p* = 0.51	*β* = −0.001, *p* = 0.99	*β* = 0.001, *p* = 0.69
RR‐SBP TE	*β* = 0.01, *p* = 0.67	*β* = 0.001, *p* = 0.95	*β* = 0.12, *p* = 0.13

*Note:* HTN, hypertensive; NTN, normotensive; PWV, pulse wave velocity; RR, R‐R interval; SBP, systolic blood pressure; TE, transfer entropy.

* *p* and *β* values were obtained by using linear regression analysis.

**FIGURE 1:**
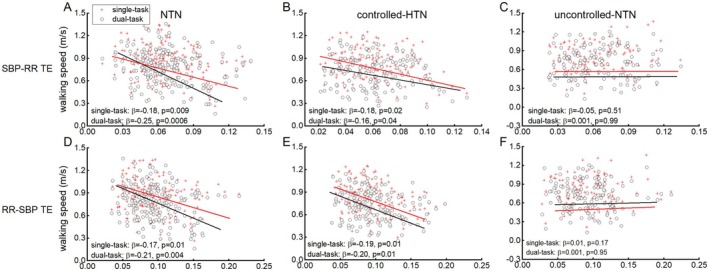
The associations of SBP‐RR (A–C) and RR‐SBP (D–F) TE and single‐ and dual‐task walking speed in groups of NTN, controlled‐HTN and uncontrolled‐HTN. The linear regression models demonstrated that in NTN and controlled‐HTN groups, participants with greater SBP‐RR and/or RR‐SBP TE had slower walking speed in both single‐ and dual‐task conditions (*β* = −0.25 to −0.16, *p* = 0.0006–0.04). Such associations were not observed in uncontrolled‐HTN group (*β* = −0.05 to 0.01, *p* > 0.17).

For walking speed, the linear regression models demonstrated that: in the NTN group, participants with greater SBP‐RR and/or RR‐SBP TE had significantly slower walking speed in single‐ and dual‐task conditions (*β* = −0.25 to −0.17, *p* = 0.0006–0.01); in the controlled‐HTN group, similar results were observed, that is, participants with greater SBP‐RR and/or RR‐SBP TE were associated with slower walking speed (*β* = −0.20 to −0.16, *p* = 0.01–0.04); while no such significant associations between TE and walking speed were observed in the uncontrolled‐HTN group (*β* = −0.05 to 0.01, *p* > 0.51) (Figure 1, Table 2).

**FIGURE 2:**
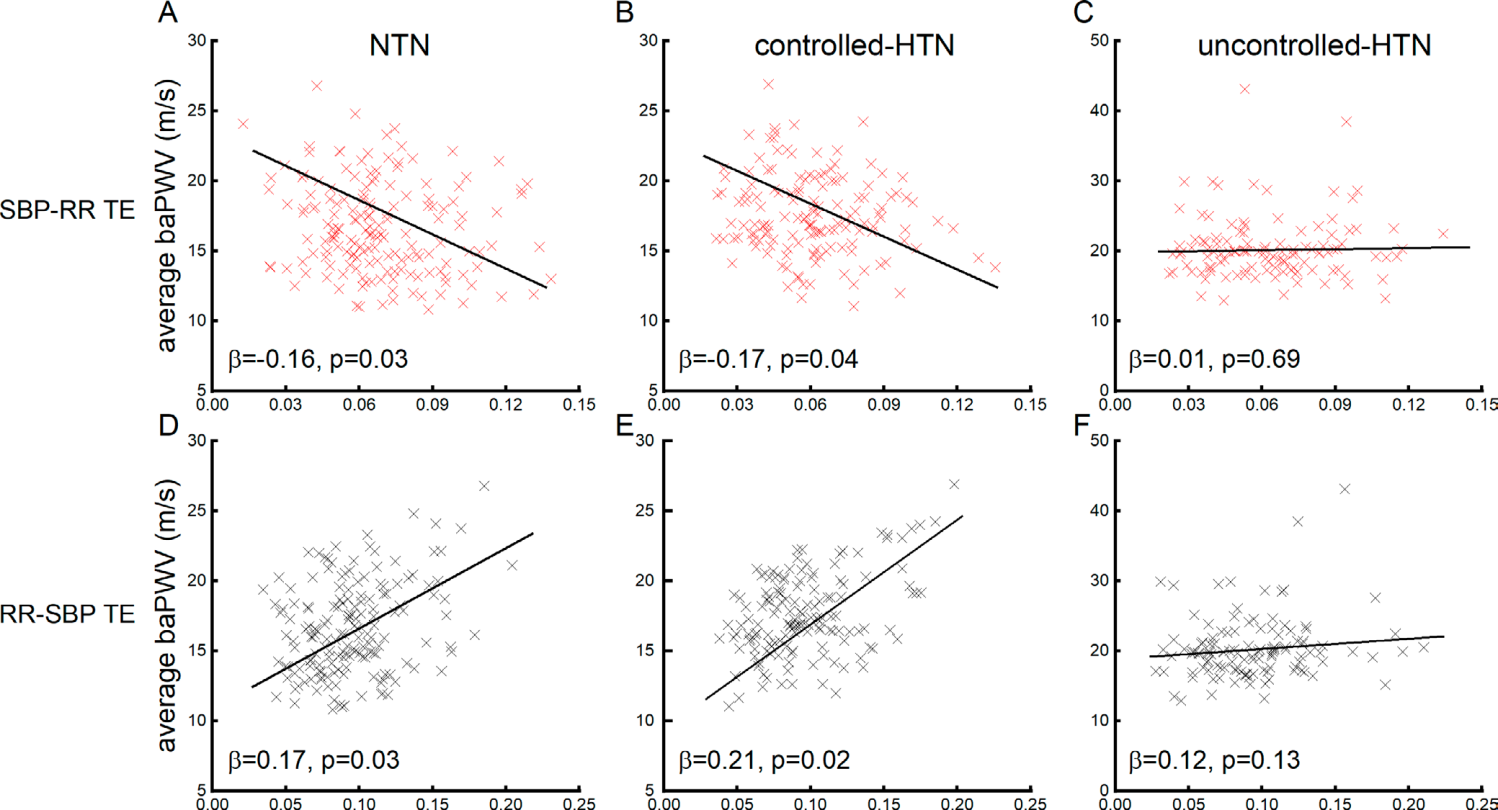
The associations of SBP‐RR (A–C) and RR‐SBP (D–F) TE and average baPWV in groups of NTN, controlled‐HTN, and uncontrolled‐HTN. The linear regression models demonstrated that in NTN and controlled‐HTN groups, participants with greater SBP‐RR TE had lower average baPWV (*β* = −0.17 to 0.16, *p* < 0.04); but those with greater RR‐SBP TE had greater average baPWV (*β* = 0.17–0.21, *p* < 0.03). No such associations were observed in the uncontrolled‐HTN group (*β* = 0.01–0.12, *p* > 0.13).

For PWV, the linear regression models demonstrated that: in NTN group, participants with greater SBP‐RR (*β* = −0.16, *p* = 0.02) and/or lower RR‐SBP TE (*β* = 0.17, *p* = 0.03) had significantly lower average baPWV; in controlled‐HTN group, similar results were observed, that is, participants who were with greater SBP‐RR (*β* = −0.17, *p* = 0.04) and/or lower RR‐SBP TE (*β* = 0.21, *p* = 0.02) had lower average baPWV; while in uncontrolled‐HTN group, no such significant associations were observed between TE and average baPWV (*β* = 0.001–0.12, *p* > 0.13) (Figure 2, Table 2).

The results of TE between DBP and RR (i.e., DBP‐RR TE and RR‐DBP TE) were similar and presented in Table S2.

## 4 Discussion

To our knowledge, this is the first study using transfer entropy to understand bidirectional information transfer between the regulation of heartbeat and blood pressure in the cardiovascular system based upon a clinical dataset of older adults. Our observations reveal hypertension‐related alterations in such bidirectional interactions between cardiac and vascular dynamics at resting state and seemingly “paradoxical” associations between the transfer entropy, walking speed and arterial stiffness among groups of NTN, controlled‐HTN and uncontrolled‐HTN. These findings provide novel insights into non‐linear interaction between the cardiac vascular regulatory processes, and such directional information transfer between them may capture vascular health, as well as the compensatory mechanisms through which functional performance (e.g., walking) can be maintained when the cardiovascular system is affected by age‐related conditions (i.e., hypertension).

In NTN group, the TE is greater in both directions (i.e., BP‐RR and RR‐BP) compared to those with HTN (both controlled and uncontrolled HTN), indicating a well‐coupled cardiovascular system in which heartbeat dynamics reliably drive blood pressure fluctuations, while blood pressure fluctuations, in turn, regulate heartbeat through a robust baroreflex mechanism (DeBoer et al. 1987). This is consistent with previous work showing that in healthy participants with intact autonomic control, the directed information flow between heart rate variability and blood pressure regulation, as measured by conditional Granger causality, was greater, suggesting balanced interplay provides adequate resilience against perturbations and stressors (Faes et al. 2011; Porta and Faes 2013). The bidirectional high TEs here thus reflect an adaptive closed‐loop regulatory procedure in the NTN group that the heart and vessels exchange information efficiently to maintain homeostasis of cardiovascular regulation, enabling the capacity of the individuals to successfully maintain functional performance in everyday life.

In contrast, lower TEs in both directions are observed in both uncontrolled and controlled‐HTN, suggesting the disrupted cardiovascular communication. In HTN, low levels of TE from blood pressure to heartbeat indicate well‐documented blunted baroreflex sensitivity, such that the blood pressure fluctuations cannot sufficiently influence heartbeat through autonomic reflexes (Griendling et al. 2021; Jones et al. 2003; Grassi et al. 1998; Parati et al. 2000; La Rovere et al. 2008). The low level of TE from heartbeat to blood pressure reflected an altered feedforward mechanical transmission from RR timing into BP. This RR‐BP pathway captures not only the mechanical contribution of ventricular stroke volume, but also arterial compliance, peripheral resistance, and diastolic runoff (Porta et al. 2015, 2013). Although arterial stiffening in HTN can theoretically increase the mechanical sensitivity of BP to cardiac timing by accelerating diastolic pressure decay, multiple coexisting impairments in hypertension—including reduced arterial reservoir function, altered ventricular–vascular coupling, and autonomic dysregulation—can collectively reduce the directional coupling from RR to BP. Thus, even in the presence of vascular stiffening, the overall feedforward RR‐BP TE may still decline in our hypertensive cohorts. Interestingly, in the controlled‐HTN group, lower TEs are observed. This may suggest that anti‐hypertensive medication appears to dampen the direct influence of cardiac variability on blood pressure, lowering feedforward RR‐BP TE (Schettini et al. 2023), while baroreflex sensitivity remains insufficiently restored (Grassi et al. 2006), resulting in persistently low feedback BP‐RR TE. This interpretation aligns with prior reports that pharmacological blood pressure control improves hemodynamic stability but does not fully normalize autonomic reflex regulation (Grassi et al. 1998; Berdeaux and Giudicelli 1987). These observations support the interpretation that cardiovascular health is marked by robust reciprocal interactions: controlled hypertension by generalized dampening, and uncontrolled hypertension by severely diminished information transfer reflecting regulatory breakdown.

Our results revealed distinct and seemingly “paradoxical” associations between BP‐RR TE and both mobility and vascular measures within each group. In both NTN and controlled‐HTN, higher TE in either direction was consistently associated with slower walking speed. This indicates that individuals with diminished mobility may engage greater cardiovascular coupling—both feedback (BP‐RR TE) and feedforward (RR‐BP TE)—as a compensatory strategy to stabilize hemodynamics during the ambulation of walking (Parati et al. 2019; Silvani et al. 2017). A potential greater association between TEs and dual‐task walking was observed compared to that of single‐task walking, indicating that such compensatory cardiovascular engagement is amplified when cognitive load challenges the regulation of walking. In contrast, these relationships were absent in uncontrolled‐HTN, reflecting the loss of adaptive scaling capacity once baroreflex pathways are severely blunted in this cohort (Hall et al. 2012). The associations between TE and arterial stiffness (as measured by baPWV) reveal opposite directional patterns. Specifically, higher BP‐RR TE is associated with lower baPWV, indicating that elastic arteries enhance baroreceptor activation and reflex‐driven modulation of heartbeat (Bank et al. 1995). Conversely, higher RR‐BP TE is associated with higher baPWV, indicating that stiffened arteries accelerate diastolic runoff, increasing the mechanical sensitivity of BP to heartbeat fluctuations and amplifying the direct transmission of heartbeat fluctuations into blood pressure fluctuation (Schiffrin 2004). The persistence of these associations in controlled‐HTN, albeit attenuated, indicates that TE remains sensitive to vascular mechanics even when blood pressure is pharmacologically stabilized, while their absence in uncontrolled‐HTN highlights the collapse of this regulatory interplay.

Therefore, these observed seemingly “paradoxical” associations suggest that the bidirectional TE simultaneously captures different facets of cardiovascular regulation. With respect to baPWV, TE captures two physiological dimensions, that is, the baroreflex competency (via BP‐RR TE) and the integrity of mechanical ventricular–vascular coupling (via RR‐BP TE) (Regnault et al. 2024). With respect to mobility, TE reflects functional reserve/compensation of the cardiovascular system, whereby slower walkers upregulate cardiovascular coupling to maintain stability under stress (i.e., dual‐task condition) (Novak et al. 2007). Therefore, characterizing TE between heartbeat and BP at resting state provides novel insight into cardiovascular hemodynamics and into how vascular health, autonomic regulation, and functional performance interact across the spectrum of health and disease.

Additionally, the traditional metrics of the cardiovascular system (e.g., mean blood pressure level and standard heart rate variability) provide valuable insights, while often overlooking the underlying nonlinear dynamics of coupling between heartbeat and blood pressure fluctuation. Transfer entropy, by quantifying direction‐specific information flow, may help characterize the diminished baroreflex function, exaggerated feedforward cardiac influence, or overly dampened dynamics resulting from pharmacological treatment. Moreover, TE profiling may aid in monitoring the effects of anti‐hypertensive therapies and guiding personalized interventions aimed not only at lowering blood pressure but also at preserving or restoring physiological dynamic interactions. More broadly, our results suggest that while TE among physiological systems can be a measure of health state, optimal levels of TE are likely to exist for each relationship and under different conditions. This implies that we cannot simply characterize high TE as “good” or “bad,” but rather that context‐specific optima will need to be identified, and that deviations in either direction from these homeostatic norms may indicate impaired physiology.

Several limitations should be noted. This work consisted of outpatient older adults who were relatively healthy without overt conditions, and the BP and RR were measured during resting state. Previous studies have also characterized the TE between BP and RR during the physiological challenges (orthostatic tilt test, general anesthesia) (Porta et al. 2015, 2013). For example, Porta and colleagues demonstrated in one study that during the orthostatic tilt test, the joint TE (JTE) and conditional JTE (CJTE) from BP to RR were decreased, but the RR‐BP JTE and/or CJTE were increased, along with the increase of orthostatic challenge magnitude in healthy adults (age: 21 to 48 years) (Porta et al. 2015). Therefore, future work consisting of other older adult populations (e.g., older adults with heart failure or orthostatic hypotension) with the measurement of BP and RR during physiological challenges is needed to examine the generalizability of the findings in this work and to explore the clinical significance of TE beyond the observations in this study. The embedding dimensions (i.e., value of k and l) were determined by using uniform analyses here (Faes et al. 2008). A nonuniform multivariate embedding strategy has also been proposed, enabling protection against over‐embedding and avoiding redundancy and irrelevant results (Porta et al. 2014). Therefore, it would be worthwhile to implement such methods in future work with longer data that require more cautious selection of the embedding dimension. This work is based upon cross‐sectional data, though TE may provide the causal relationship between two procedures. Future studies with longitudinal design are needed to explore the relationship between the change of TE and that of cardiovascular health and functional performance in the aging process and age‐related conditions. We here only focused on walking speed and arterial stiffness; future work is needed to explore the relationship between this bidirectional information transfer of the cardiovascular system and other important functions, such as cognitive performance, and other important characteristics of cardiovascular health. This may ultimately provide a more comprehensive understanding of the nonlinear coupled regulation in the cardiovascular system. Nevertheless, this work demonstrates the potential of using TE as a sensitive marker of cardiovascular regulation, which is worth being considered and incorporated in the cardiovascular assessment, providing clinically meaningful insights into the complex regulatory networks of cardiovascular systems.

## Author Contributions

Study design: X.J. and J.Z.; data collection: X.J., H.R., M.L., D.Z., S.L., Z.L., J.Y., and N.X.; data analysis: X.J., H.R., A.A.C., S.P., and J.Z.; literature search: X.J., H.L., H.R., M.L., S.L., A.A.C., and S.P.; data interpretation and manuscript preparation: all authors.

## Funding

This work was supported by Research Program of Central Health Commission (No. 2024YB59); Shenzhen Science and Technology Program (JCYJ20240813103817024); Shenzhen People’s Hospital Physician Scientist Training “Five Three Program” (Grant No. SYWGSLCYJ202204); Shenzhen Science and Technology Research and Development Fund for Sustainable development project (KCXFZ20201221173411032); and National Natural Science Foundation of China (NSFC82371471).

## Conflicts of Interest

The authors declare no conflicts of interest.

## Supporting information


**Table S1:** The conditional entropy of embedding dimensions from 1 to 7.
**Table S2:** The association between transfer entropy between heartbeat and DBP and walking performance and arterial stiffness*.

## Data Availability

The data that support the findings of this study are available on request from the corresponding author. The data are not publicly available due to privacy or ethical restrictions.
