# Supplementary material for: The Disrupted Bidirectional Regulation and Coupling of Resting‐State Blood Pressure and Heartbeat in Hypertension
Source: Aging Cell. 2025 Dec 26;25(1):e70338. doi: 10.1111/acel.70338 (PMC12741931; doi:10.1111/acel.70338)
Supplement: Supplementary file 1 — Table S1: The conditional entropy of embedding dimensions from 1 to 7. Table S2: The association between transfer entropy between heartbeat and DBP and walking performance and arterial stiffness*. [file ACEL-25-e70338-s001.docx]

**Supplementary materials**

**The determination of optimal embedding dimension for the calculation of transfer entropy.**

To determine the appropriate embedding dimension (i.e., the value of k and k) for the calculation of bidirectional TE between RR and BP (i.e., both SBP and DBP), we performed the computation following the recommendation as described in previous work.^46^ Specifically, the criteria of optimal embedded dimension were defined as the smallest value of k and l that provided lowest prediction uncertainty (i.e., no more information gain) as measured by smallest conditional entropy of RR and BP. We computed the conditional entropy for each of the four directional relationships (i.e., RR-SBP, RR-DBP, SBP-RR, and DBP-RR) when using k=l=1 to k=l=7. The results were presented in Supplementary Materials (Table S1). Highly consistent pattern was observed, that is, the conditional entropy for all the directional relationships decreased steeply from k=l=1 to k=l=3; from k=l=3 to k=l=4, the reduction in conditional entropy became small and diminishing; and when k=l>4, such conditional entropy was minimal, indicating that predictive information is nearly saturated and the over-embedding existed.

Meanwhile, the beat-to-beat cardiovascular coupling processes (e.g., baroreflex feedback, mechanical RR-BP transmission, arterial reservoir effects) unfolded over short memory episodes of approximately 1–3 beats and adding more past samples may potentially add statistical noise without significantly additional physiologically meaningful information. Additionally, considering the potential risk of over embedding bias by increasing embedding that may substantially enlarge the state space (e.g., 3⁷=2,187 states when k=l=3 and 3⁹=19,683 when k=l=4),^46^ we thus chose k=3 and l=3 for the calculation of RR-SBP and -DBP TE, and SBP- and DBP-RR TE.

**The information exchange between DBP and heartbeat regulation**

For the directional information exchange from DBP to heartbeat, the one-way ANOVA models demonstrated significant effects of group on DBP-RR TE (p=0.02), and such effects were independent from age, sex, and BMI. Specifically, the post-hoc analysis revealed that compared to NTN group, the TEs in both controlled- (p=0.002) and uncontrolled-HTN (p=0.0005) were significantly lower; and no significant differences between controlled- and uncontrolled-HTN groups were observed (p=0.56) (Table 1).

For the directional information exchange from heartbeat to DBP, the ANOVA models demonstrated significant effects of group on RR-DBP TE (p=0.01), and such effects were independent from age, sex, and BMI. Specifically, the post-hoc analysis revealed that compared to NTN group, the TE in controlled-HTN group (p=0.007), and in uncontrolled-HTN group (p=0.01), was significantly lower; and no significant differences between controlled- and uncontrolled-HTN groups were observed (p=0.92) (Table 1).

Table S1. The conditional entropy of embedding dimensions from 1 to 7.

|  | Embedding dimension | | | | | | |
| --- | --- | --- | --- | --- | --- | --- | --- |
|  | 1 | 2 | 3 | 4 | 5 | 6 | 7 |
| RR-SBP | 0.98±0.09 | 0.78±0.11 | 0.55±0.11 | 0.45±0.11 | 0.11±0.08 | 0.06±0.05 | 0.03±0.03 |
| RR-DBP | 0.82±0.18 | 072±0.15 | 0.50±0.11 | 0.47±0.11 | 0.10±0.10 | 0.08±0.07 | 0.04±0.05 |
| SBP-RR | 0.86±0.18 | 0.73±0.15 | 0.52±0.09 | 0.46±0.08 | 0.11±0.06 | 0.06±0.04 | 0.03±0.03 |
| DBP-RR | 0.87±0.18 | 0.76±0.15 | 0.56±0.12 | 0.42±0.12 | 0.12±0.10 | 0.08±0.07 | 0.04±0.04 |

RR: R-R interval; SBP: systolic blood pressure, DBP: diastolic blood pressure.

Table S2. The association between transfer entropy between heartbeat and DBP and walking performance and arterial stiffness*.

|  | walking speed | | average baPWV |
| --- | --- | --- | --- |
|  | single-task | dual-task |  |
| NTN | | | |
| DBP-RR TE | β=-0.19, p=0.007 | β=-0.21, p=0.002 | β=-0.18; p=0.02 |
| RR-DBP TE | β=-0.19, p=0.007 | β=-0.17, p=0.02 | β=0.19; p=0.0003 |
| controlled-HTN | | | |
| DBP-RR TE | β=-0.16, p=0.05 | β=-0.13, p=0.09 | β=-0.15; p=0.06 |
| RR-DBP TE | β=-0.19, p=0.02 | β=-0.16, p=0.03 | β=0.23; p=0.01 |
| uncontrolled-HTN | | | |
| DBP-RR TE | β=0.07, p=0.41 | β=-0.001, p=0.95 | β=-0.04; p=0.59 |
| RR-DBP TE | β=0.001, p=0.90 | β=0.01, p=0.61 | β =0.001; p=0.82 |

NTN: normotensive HTN: hypertensive; PWV: pulse wave velocity; RR: R-R interval; SBP: systolic blood pressure, DBP: diastolic blood pressure; TE: transfer entropy.

*: the p and β values were obtained by using linear regression analysis.
